# Lactococcus lactis, A Tool for the Delivery of Therapeutic Proteins Treatment of IBD

**DOI:** 10.1100/tsw.2001.37

**Published:** 2001-05-11

**Authors:** Lothar Steidler

**Affiliations:** Department of Molecular Biology, VIB, Ghent University, Ghent, Belgium

**Keywords:** Lactococcus Lactis, Interleuken-10, IBD

## Abstract

Inflammatory bowel disease (IBD) is a group of chronic intestinal inflammatory diseases that consists of ulcerative colitis (UC), an inflammation of the large intestine, and Crohn's disease (CD), which can affect any part of the gastrointestinal tract. IBD affects approximately 1 in every 1000 individuals in western countries. There is a marked tendency in the age of onset toward gradually younger people. IBD represents a genuine problem in public health because of the absence of etiologic treatment. The clinical image is characterized by recurrent segmental or total inflammatory involvement of the large and/or small intestine, often resulting in a chronic, unpredictable course. The symptoms of both are extremely unpleasant and impact all aspects of quality of life. They include diarrhea, abdominal pain, rectal bleeding, fever, nausea, weight loss, lethargy, and loss of appetite. If left untreated, malnutrition, dehydration, and anemia follow, which, in extreme cases, can even lead to death. Although many patients are managed successfully with conventional medical therapy, such as anti-inflammatory corticosteroid treatment, some stay refractory to treatment, most will have recurrent activity of disease, and two thirds will require surgery. Administered orally or by injection, only a fraction of the active components of most conventional drugs reaches the intended target site, the inflamed intestinal lining. This is not only an inefficient way to deliver drugs, but, more important, means that patients are often subject to a spectrum of unpleasant side effects that result from the high levels of the drugs in other, otherwise healthy tissues and organs of the body.

Our research group at the VIB (Flanders Interuniversitary Institute for Biotechniology) has developed a novel approach for the treatment of chronic intestinal inflammation in mice that has the potential to revolutionise the treatment of IBD. We have genetically modified *Lactococcus lactis*, a bacterium widely used in the manufacture of dairy products, to produce a natural anti-inflammatory, *interleukin-10* (IL-10). When administered orally this novel strain can cure and prevent animal models of inflammatory bowel disease in mice by releasing IL-10 into the intestine. These results were published in the August 25$^{th}$ 2000 issue of *Science* [1].

*Lactococcus lactis* is a noninvasive and noncolonising Gram positive food grade bacterium. Its main use lies in the manufacture of fermented milk, vegetable, and meat produce. The earliest records of cheese making date from approximately 10,000 years back. At no point during this period of extensive consumption has *L. lactis* ever been associated with any form of pathology. Neither has any risk factor been reported for particular subpopulations, such as infants or elderly people, in association with the intake of this microorganism. It is therefore considered a "generally regarded as safe" (GRAS) microorganism by the U.S. FDA.

We [2,3] first reported on the use of recombinant *L. lactis* for the production of cytokines of both murine and human origin. Surprisingly, continuous synthesis of these cytokines did not influence the growth rate of the organism. This lead to the idea that these strains might be adequate tools for the *in vivo* delivery of cytokines, in particular at mucosal surfaces. This idea showed promising as the efficient delivery of either murine interleukin-2 (mIL-2) or mIL-6 to the nasal mucosa of mice could be demonstrated [4].

To further investigate the potential of *L. lactis* as a delivery tool, we addressed the question whether a cytokine-secreting strain, administered by the oral route, could be effective in reversing the inflammation associated with experimental colitis — serving as models for the study of human IBD — in mice. As a therapeutic tool we chose mIL-10. IL-10 is produced by the body's immune cells and acts to suppress inflammation. Mice in which the IL-10 gene had been disrupted (IL-10 KO) cannot make IL-10 and spontaneously develop IBD, suggesting an important role for this natural anti-inflammatory in protection against intestinal inflammation. IL-10 is therefore a good candidate as therapy in the treatment of IBD. However, when administered by injection, the high levels of IL-10 that are distributed throughout the body can lead to a number of side effects and so IL-10 is no better in this respect, as conventional synthetic immunosuppressive therapies. By looking at the histology of the colon, we investigated the effect of feeding the recombinant mIL-10–producing strain to mice that served as IBD models. Mice in which IBD had been chemically induced could be cured by the daily feeding of mIL-10 producer *L. lactis*. Our team showed that the mechanism of action involved the active production and secretion of mIL-10 in the intestine. We also found that, compared to doses adminstered by injection, doses of 10,000-fold less IL-10 were effective. Also, the onset of IBD in IL-10 KO mice could be prevented when they were fed the *L. lactis* mIL-10 producer from the moment of weaning on.

Life-long intake of anti-inflammatory drugs is very often the fate of IBD patients. This implies that especially these pharmaceuticals should be low in risk for side effects, easy to take in, and preferably inexpensive. Lactococcal-mediated delivery can considerably reduce the amount of therapeutic. This may answer the question for sustained and localised presence of IL-10 at concentrations higher than achievable or even desirable, with regard to latent side effects, when given through systemic delivery. Since *L. lactis* is a noncolonising microorganism, accurate dosage and timing during treatment can easily be obtained. This method is effective via the oral route, which is by far the most desirable for pharmacological formulations. Moreover, lactococci can be cultivated on cheap growth media so the method is intrinsically very cost effective. It will be interesting to see through which mechanism of action this novel therapeutic strategy exerts its beneficial effects on the immune system and whether it will also show effective in humans.

One could speculate that a vast multitude of protein therapeutics — not necessarily restricted to cytokines — could be delivered through *L. lactis*. The spectrum of activities would therefore not need to be limited to intestinal inflammatory disorders but could be as broad as all diseases treatable through mucosal application of protein drugs.

| | | |
|---|---|---|
| Received: | February | 15, 2001 |
| Accepted: | March | 26, 2001 |
| Published: | May | 11, 2001 |
